# Anti-ovarian tumor response of donor peripheral blood mononuclear cells is due to infiltrating cytotoxic NK cells

**DOI:** 10.18632/oncotarget.6939

**Published:** 2016-01-18

**Authors:** Veethika Pandey, Jeremiah L. Oyer, Robert Y. Igarashi, Sarah B. Gitto, Alicja J. Copik, Deborah A. Altomare

**Affiliations:** ^1^ Burnett School of Biomedical Sciences, College of Medicine, University of Central Florida, Orlando, FL, USA

**Keywords:** preclinical, adoptive cell transfer, natural killer cells, allogeneic peripheral blood mononuclear cells

## Abstract

Treatment of ovarian cancer, a leading cause of gynecological malignancy, has good initial efficacy with surgery and platinum/taxane-based chemotherapy, but poor long-term survival in patients. Inferior long-term prognosis is attributed to intraperitoneal spreading, relapse and ineffective alternate therapies. Adoptive cell therapy is promising for tumor remission, although logistical concerns impede widespread implementation. In this study, healthy PBMCs were used to examine the immune response in a mouse model with human ovarian cancer, where natural killer (NK) cells were found to be the effector cells that elicited an anti-tumor response. Presence of tumor was found to stimulate NK cell expansion in mice treated intraperitoneally with PBMC+Interleukin-2 (IL-2), as compared to no expansion in non-tumor-bearing mice given the same treatment. PBMC+IL-2 treated mice exhibiting NK cell expansion had complete tumor remission. To validate NK cell mediated anti-tumor response, the intratumoral presence of NK cells and their cytotoxicity was confirmed by immunohistochemistry and granzyme activity of NK cells recovered from the tumor. Collectively, this study highlights the significance of NK cell-cytotoxic response to tumor, which may be attributed to interacting immune cell types in the PBMC population, as opposed to clinically used isolated NK cells showing lack of anti-tumor efficacy in ovarian cancer patients.

## INTRODUCTION

Ovarian cancer is the second leading gynecological cancer [1]. Although current chemotherapy using carboplatin-paclitaxel is effective as a first-line therapy, 70% of patients with advanced disease succumb to tumor relapse within less than five years, despite initial response to chemotherapy [2]. Thus, there is an urgency to develop strategies for prevention of tumor relapse and for increasing overall patient survival. The immune system is protective against ovarian cancer and thus, adjuvant immunotherapy post-surgery and with chemotherapeutics could be effective for preventing relapse and extending survival [3, 4]. Studies show the link between cancer stem cells and ovarian tumor recurrence, and that treatment with chemotherapeutics selects resistant tumor populations causing increased risk of tumor relapse [5, 6]. Such resistant tumor cell populations are susceptible to lymphocyte-mediated lysis, particularly by NK cells [7]. Therefore, NK cell-based adjuvant immunotherapy could have significant impact on sustaining remission and improving overall treatment efficacy.

Immunotherapy has impacted treatment of multiple cancers. Monoclonal antibody-based approaches are beneficial for long-term effects, but a challenge is potential escape by tumors [8]. Adoptive cell therapy (ACT) with various immune cell types, T cells, dendritic cells (DCs) or natural killer (NK) cells in clinical trials hold promise. A landmark publication in 2003 showed that the infiltration of T cells into ovarian tumors improved survival [4]. Since tumor infiltrating immune cells influence the severity and overall disease outcome, attempts have been made to adoptively transfer tumor infiltrating lymphocytes (TILs) after lymphodepletion with successful results in metastatic melanoma patients [9, 10]. DCs can activate the adaptive immune system for a robust response against infection or against transformed cells, and trials using DCs are ongoing (Clinicaltrials.gov identifier: NCT01875653). DCs function as antigen presenting cells for naïve CD4 and CD8 T cells [11]. To circumvent the need for T cell “education”, chimeric antigen receptor (CAR)-T methods can genetically modify patient T cells to express a targeting receptor for a tumor specific antigen. In contrast to naïve CD4 and CD8 T cells that need to be “educated” or engineered *ex vivo*, innate NK cells are naturally cytotoxic towards malignant cells [12]. *In vitro* studies show that resting NK cells from healthy donors target isolated tumor cells from the peritoneal ascites of ovarian carcinoma patients [13]. In this respect, ACT using cytolytic NK cells for cancer treatment is more advantageous since NK cells do not require prior sensitization with an antigen and are not limited to targeting only tumors that have a specific marker as in CAR-T methods [14].

Clinical studies for ovarian and breast cancer using intravenously (IV) delivered NK cells enriched by CD3 depletion of PBMCs from haploidentical donors failed to show *in vivo* NK cell expansion perhaps due to suppression by host regulatory T (Treg) cells or myeloid-derived suppressor cells [15]. Therefore, there is still an insufficient understanding about factors required for NK cell expansion and *in vivo* persistence for successful clinical outcome. A previous pre-clinical study showed that intraperitoneally (IP) delivered enriched NK cells could have anti-tumor response against ovarian cancer, and that NK cell cytotoxicity may be affected by the mode of delivery that could bypass hurdles of NK cell homing to the tumor location [16].

The quality of immune response to ovarian cancer has a significant impact on disease prognosis [17–19]. In the context of a complete immune system, innate NK cells can have direct cytotoxicity towards transformed cells as well as interact with DCs to induce IFN-γ production which primes Th1 cells [20] and further enhances cytotoxic T cell responses [21]. NK cells are indispensable for effective DC-based immunotherapy, as loss of NK cells has shown to result in defective tumor immunity [22]. Such studies highlight the importance of NK cell interactions with both, innate and adaptive immune cell types, to affect adaptive immunity for effective anti-tumor response.

Here, we examine NK and T cells' response to tumor as part of an unbiased whole PBMC population as opposed to treating with selectively enriched NK or T cell populations. The study examines the kinetics of effector subtypes involved in the acute anti-tumor response of innate and adaptive components of PBMCs and identifies NK cells as the main effector cell of PBMCs' response, acting as a first line of anti-tumor defense. It also highlights the importance and points to the need for further studies to delineate other interacting immune cell types to strategically employ them as an adjuvant regimen for a safe and effective NK cell-based immunotherapeutic approach.

## RESULTS

### Treatment with unselected healthy PBMCs clears human ovarian tumors engrafted in mice

**Figure 1:**
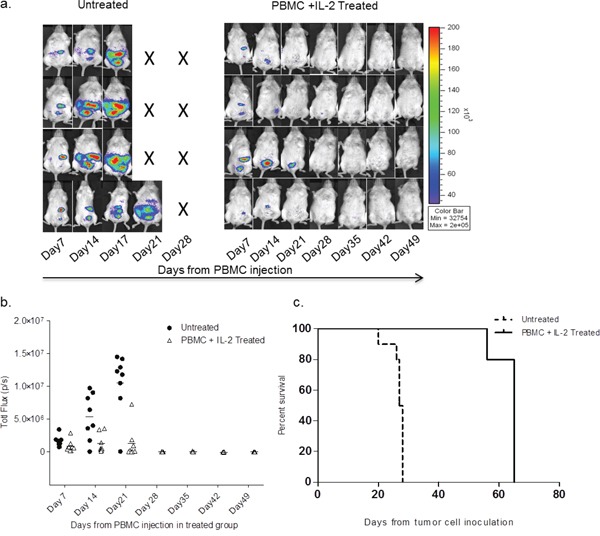
SKOV-3/GFP-Luc tumor bearing mice show complete remission of tumor upon healthy human PBMC and IL-2 treatment **a.** Bioluminescence of representative mice showing SKOV-3/GFP-Luc tumor burden over 7 weeks. Mice shown in the left panel were untreated, whereas the ones in the right panel were treated with PBMCs+IL-2 and monitored for tumor growth. **b.** Quantification of the tumor signal (total flux) obtained from imaging. P-value was <0.001 for the first three time-points (two-way ANOVA, GraphPad Prism). **c.** Curve showing the difference in overall survival of the untreated and PBMC treated mice from the day of tumor cell inoculation. p= 0.001 (Log-rank test, GraphPad Prism).

The interplay among multiple immune cell types in response to the presence of a tumor is complex and is still poorly understood. To address the therapeutic effectiveness of unselected immune cells from normal donor PBMCs in response to the presence of tumor, NSG mice that were IP inoculated with 1 × 10^6^ SKOV-3/GFP-Luc cells were monitored for engraftment. Mice that showed engraftment 7 days post inoculation were then treated with human ‘PBMC+IL-2’ (IL-2 dose: 1,000 U thrice weekly) or remained ‘untreated’. Another group of non-tumor bearing mice was injected with PBMC+IL-2 as a control. Treatment effectiveness was assessed by monitoring tumor size and overall health for 7 weeks after starting treatments. Serial imaging (Figure 1a) shows significant differences in tumor progression between the ‘untreated’ and the ‘PBMC+IL-2 treated’ groups. Untreated mice succumbed to disease in ~3 weeks, whereas tumor-engrafted mice treated with PBMC+IL-2 showed reducing tumor burden. Figure 1a shows reduction of tumor size in the treated group and total luminescence flux from the peritoneal tumor images acquired after PBMC injections (Figure 1b) also demonstrates the effect of treatment compared with no treatment (p=0.0003, two-way ANOVA). A clear survival difference was observed in PBMC-treated compared to untreated mice (p=0.001, Log-rank Test) (Figure 1c). Untreated mice were euthanized upon health deterioration including abdominal bloating due to ascites and hunched posture. Overall, tumor-engrafted mice treated IP with whole PBMCs showed tumor regression within 3 weeks of PBMC treatment as compared to the untreated mice that succumbed to the disease within the same time frame.

### Expansion of NK cells in peripheral blood and peritoneal cavity of PBMC treated tumor-engrafted mice

**Figure 2:**
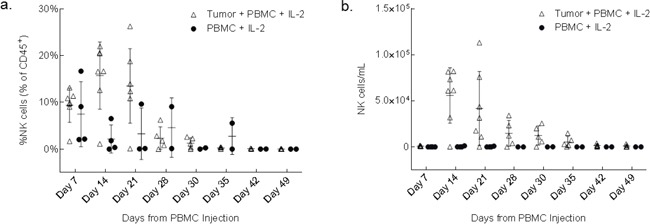
NK cells increase in peripheral blood in response to the presence of engrafted tumor Plots show the fraction of human lymphocytes **a.** and concentration **b.** of human CD45^+^, CD56^+^, CD3^−^ NK cells in mouse peripheral blood at the indicated number of days after initiation of treatment. p=0.005 (left); p= 0.009 (right) (two-way ANOVA, GraphPad Prism).

**Figure 3:**
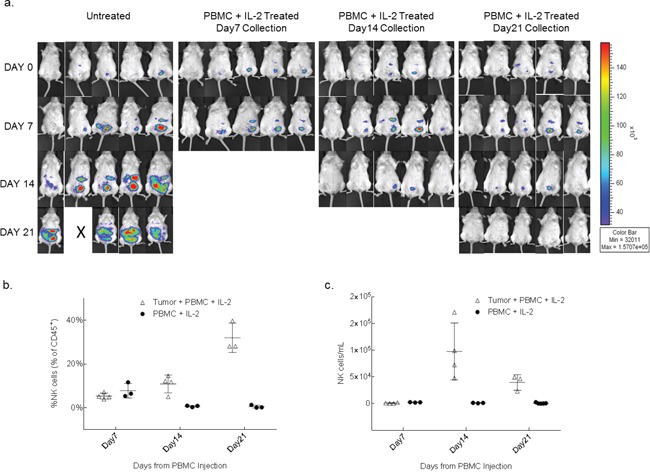
NK cells from whole PBMCs expand in the presence of tumor within the peritoneal cavity **a.** After 10 days of SKOV-3/GFP-Luc engraftment, 3 groups were treated with 1×10^6^ human PBMC+IL-2. Five mice were euthanized every week for 3 weeks for the analysis of peritoneal cavity wash. Another 3 groups of mice (not shown here) with no tumor and injected only with PBMCs+IL-2 were also collected along with the tumor bearing mice, as controls. **b.** Plots show the trend of expansion and reduction of NK cells in the peritoneal cavity expressed as fraction of human lymphocytes and **c.** as concentration over a time period of 3 weeks. p=0.0001 (b); p=0.0052 (c) (two-way ANOVA, GraphPad Prism).

To determine if the cytolytic effector components in the anti-tumor response with the PBMC+IL-2 treatment is due to a single or synergistic lymphocyte populations in the allografted PBMCs, levels of immune cells were serially tracked in mouse peripheral blood for up to 7 weeks after PBMC injection. Delivering unselected healthy donor PBMCs along with low-dose IL-2, the expansion of NK cells was observed in the blood of only mice seeded with tumor. The Tumor+PBMC+IL2 group had day 7 counts of 650 to 1600 NK cells/mL, and numbers of NK cells/mL increased significantly at the day 14 time point. The PBMC+IL2 group typically had counts <100 NK cells/mL and did not increase significantly. Also, mice that were injected only with PBMCs+IL-2 but without any tumor, showed no engraftment of NK cells (Figure 2). In a subsequent experiment, sets of mice treated with PBMC+IL-2 were collected on days 7, 14 and 21 post-treatment to examine the human lymphocyte population and their interaction with the tumor in the intraperitoneal cavity (Figure 3). NK cells/mL counts in peritoneal wash were ≤2600 NK cells/mL at day 7, after having been injected directly into the peritoneal cavity, and significantly increased at day 14. Similar to the previous experiment, treatment of tumor-seeded mice with PBMC and IL-2 delivered IP resulted in reduced tumor and complete clearance by 21 days after initiation of treatment. For each of the mice sacrificed on days 7, 14 and 21, cells were collected from the intraperitoneal cavity and were analyzed by flow cytometry. As seen previously in the analysis of peripheral blood, NK cell expansion occurred only in the presence of tumor, and despite IL-2 stimulation, mice without any tumor did not show NK cell engraftment, expansion or persistence. NK cell number peaked at day 14, when tumor signal intensity was also at its peak (Figure 1b) and then started to decline as the tumor regressed. Data from the blood analyzed during this experiment is shown in Supplemental Figure 1 with a similar trend as seen in the blood analysis of the survival experiment (Figure 2). These results show that the presence of tumor elicits specific expansion of NK cells from an unselected PBMC population, in the presence of low dose IL-2.

The result presented above suggest the anti-tumor activity of NK cells as part of a PBMC population. Follow-up experiments were conducted with either isolated NK cells or with NK cell depleted PBMCs. Supplemental Figure 6 shows a survival curve and the peripheral blood concentrations of total lymphocytes, NK cells, NKT cells and T cells from these follow-up experiments. The survival curve of the mice treated with isolated NK cells overlapped with that of the untreated mice and we did not observe any immune cell growth in the blood of the mice treated with the isolated NK cells. This suggests that isolated NK cells did not have the necessary cross-talk with other cell populations in the PBMCs, such as the availability of IL-2 secreting T cells. The exogenous IL-2 provided was not sufficient to support NK cell expansion and thus, they did not thrive long enough to show any tumor cytotoxicity. In case of the NK cell depleted-PBMCs treated group, the survival curve overlapped with that of the whole PBMCs treated group. Although there were no significant NK cells/mL numbers, at day 14 NKT cell expansion was seen in these mice (mean ~1 × 10^5^ NKT cells/mL). In previous experiments, we have observed NKT cell expansion at day 14 (mean ~1.8 × 10^4^ NKT cells/mL), but higher numbers of NKT cells/mL were observed in this experiment where NK cells were depleted.

### NK cells infiltrate tumor and show markers of cytotoxicity

**Figure 4:**
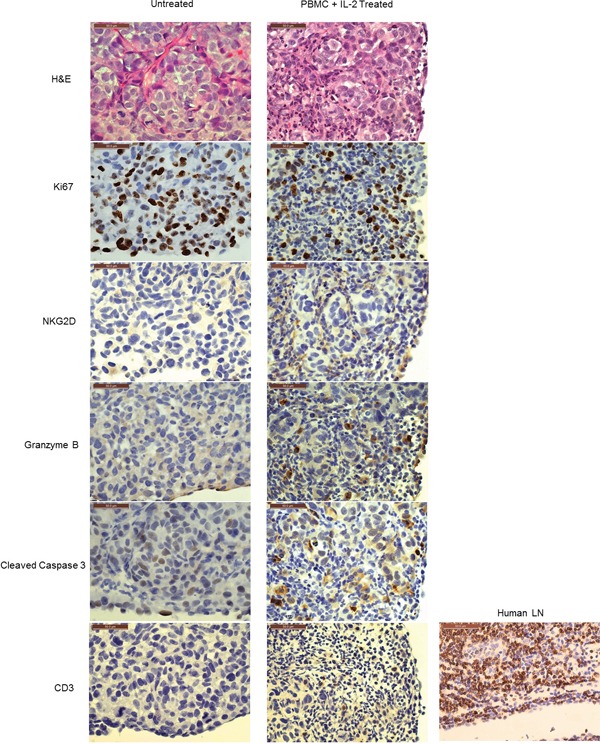
*In vivo* expanded NK cells infiltrate tumors and mediate tumor killing The left panel shows representative images of a tumor nodule from the ‘untreated’ group of animals; the right panel shows that of ‘PBMC+IL-2 treated’ ones. The H&E image in the top panel shows infiltrating immune cells compared to the untreated tumor. Tumor cells have Ki67 staining indicating proliferation. NKG2D staining shows NK cells within the tumor of the PBMC+IL-2 treated group. Granzyme B staining shows NK cell granules containing granzyme which mediate target cell lysis. Cleaved Caspase 3 staining indicates apoptosis in the tumor cells. The last panel shows the lack of CD3 staining in the same region of the tumor showing apoptosis. A human lymph node (LN) is shown as a positive control for the human CD3 antibody. All images were acquired using a 40X objective and scale bars represent a length of 50μm.

To corroborate that NK cell expansion in the presence of tumor *in vivo* and subsequent tumor remission was due to NK cell mediated cytotoxicity, shrinking tumors were collected from 14 day treated and untreated mice for analysis by histology. NKG2D was used as an NK cell marker because it is one of the main activating receptors on NK cells that recognizes various stress induced ligands on tumor cells [22]. Staining of a tumor nodule section (Day 14 after PBMC inoculation) from the PBMC+IL-2 treated group with anti-NKG2D showed positive NKG2D staining in the peripheral region of the nodule (Figure 4), which is indicative of intratumoral infiltration of NK cells. Granzyme release was observed in the same region of the tumor. This is consistent with the presence of granzyme containing cytotoxic granules released from NK cells upon tumor cell recognition to mediate apoptosis by caspase dependent pathways [23]. Detection of cleaved caspase 3 by staining in the same region as that of granzyme release confirms NK cell induced apoptosis of tumor cells (Figure 4).

Since T cell expansion was observed in the peripheral blood (Supplemental Figure 2) and the peritoneal cavity (Supplemental Figure 3), their presence was also examined by staining with anti-CD3. CD3 staining was minimal (last panel in Figure 4) as compared to NKG2D staining in the same region of the tumor or as compared to CD3 staining in a human lymph node used as a positive control, thus confirming that the cytotoxicity markers were attributable to infiltrating NK cells killing tumors cells and not T cells. The NKG2D staining appears to be dim in the region shown, perhaps because of actively dying cells in that area due to granzyme release. A different region of the tumor shows clearer NKG2D staining (Supplemental Figure 4). These results indicate that NK cells expand in the vicinity of the tumors (peritoneal cavity) and infiltrate into the small tumor nodules to cause tumor regression observed in the PBMC+IL-2 treated mice.

### NK cells show direct cytotoxicity towards tumor cells *ex vivo*

**Figure 5:**
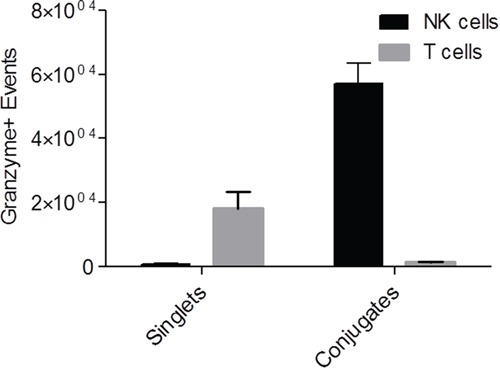
Granzyme assay showing actively cytotoxic NK cells perfused from tumors Graph representing granzyme positive events of NK and T cells present in CD45^+^ singlet or conjugated populations upon perfusion from tumors. p=0.0002 (two-way ANOVA).

To confirm that the intratumoral NK cells are cytotoxic and not anergic, intratumoral perfusions were isolated from two tumor bearing mice being treated with PBMCs+IL-2 for 14 days and were tested for granzyme activity as discussed in the methods section. Flow cytometric analysis of granzyme activity in singlet vs. doublet tumor-lymphocyte conjugate populations was performed as previously described [24], to determine the cytotoxic response of intratumoral NK and T cells against fresh SKOV-3/GFP-Luc cells (Supplemental Figure 5). Differences in scatter properties (forward scatter height vs area plots) and CD45 intensity were used to determine the conjugates (tumor cells and immune cells) and singlet cell populations. Populations were further gated for CD56^+^, CD3^−^ NK cells and CD3^+^ T cells, and for signal from granzyme activity. Figure 5 represents the events positive for granzyme activity seen in singlet and conjugate cells. Overall, significant difference (p=0.0002, two-way ANOVA) was observed in the number of granzyme-positive NK cells as compared to T cells with majority of the NK cells forming conjugates with tumor cells. These results show that isolated intratumoral NK cells are not anergic and highly cytotoxic towards SKOV-3/GFP-Luc tumor cells, and gives strong evidence that the tumor shrinkage effect observed *in vivo* is due to cytolytic activity from NK cells and not T cells.

## DISCUSSION

In this study, complete tumor remission and increased survival of tumor bearing NSG mice was observed when mice were treated with healthy donor derived PBMCs, as compared to the untreated ones. From the whole PBMC population injected, NK cell expansion was observed in the peritoneal cavity as well as in the peripheral blood of the tumor bearing mice, whereas no immune cell expansion or NK cell engraftment was seen in the control mice without any tumor. There was also a correlation between tumor shrinkage and decreasing number of NK cells in the treated mice. Importantly, flow cytometry of granzyme activity of immune cells isolated from tumors showed that 1) there were more NK cells than T cells, 2) the majority of tumor conjugates were that of tumor-NK cells and 3) these tumor-NK cell conjugates had granzyme activity. These data collectively led us to conclude that NK cells were the effector cells responsible for the tumor regression and progression-free survival of the mice.

Previous studies with either allogeneic or autologous NK cell adoptive therapy have been inconsistent in producing successful clinical outcomes due to various reasons, most importantly the inability of NK cells to expand and persist *in vivo* [25]. A phase II study with IV delivered haploidentical NK cells in ovarian and breast cancer patients with recurrent disease following lymphodepleting chemotherapy showed sub-optimal NK cell expansion [15]. Another study with adoptively transferred autologous NK cells showed proliferation and persistence *in vivo*, but the NK cells failed to exhibit cytotoxicity due to immunosuppression without any overall benefit to the health of the patients [26]. A study in a xenograft mouse model showed progress with *in vivo* persistence of NK cells by changing the mode of delivery from IV to IP, but this study required very high starting dose of *in vitro* IL-2 pre-activated NK cells (20×10^6^), enriched by T and B cell depletions of PBMCs, and high dose IL-2 (75,000 U/day IP daily for 3 weeks) in order to allow reduction in tumor burden with MA-148/GFP-Luc ovarian tumor cells [16]. In contrast, only 1 × 10^6^ whole PBMCs (8-11% NK cells) were injected IP along with very low dose of IL-2 (1,000 U thrice weekly) to induce complete SKOV-3/GFP-Luc tumor remission in this study. This suggests that a supportive or synergistic role of other cell types in the whole PBMC population is crucial for optimal NK cell cytotoxic activity against tumors. The follow-up experiment (Supplemental Figure 6) conducted with isolated NK cells or NK depleted PBMCs further supports the concept that immune cells work as a communal composition and the lack of a single cell type alters the stimulation of different immune cell type(s) as part of that composition and their potential anti-tumor response.

Cell types that are known to augment NK cell proliferation and maintenance are monocytes, DCs and T cells. Monocytes have been shown to aid in NK cell expansion mediated by some soluble factors [27]. Results from *in vitro* and clinical studies indicate that cytokines such as IL-2, 1L-12 and IL-21 enhance NK cell proliferation and effector functions such as IFN-γ secretion [28, 29]. DCs also play a role in NK cell effector functions like IFN-γ production and cytolytic activity *in vitro* [30, 31]. Mouse and human studies show that they mediate their support mainly through secretion of IL-12 [32, 33] and trans-presentation of membrane bound IL-15 on mature DCs [30, 34–36]. In addition to triggering NK cell expansion and activation through a direct cell-to-cell contact, DCs can also influence NK cells by releasing exosomes [37, 38]. A clinical study showed that vaccination with dendritic cell derived exosomes harboring NKG2D ligands and functional IL-15Rα, synergize for NK cell proliferation and activation *in vitro* and in patients [39]. In addition to monocytes and DCs, NK cells are also dependent on activated CD4^+^ T cells for the availability of IL-2, but secretion of IL-2 by these cells also causes Treg cell expansion as a negative feedback to prevent overstimulation of CD4^+^ T cells. Therefore, Tregs restrict the availability of IL-2 for NK cells causing suppression of NK cell expansion and activity [40, 41], thus validating the effect of Treg depletion to enhance tumor suppression by NK cells [42, 43]. In our study, the presence of T cells in the peritoneal cavity (Supplemental Figure 3) may have provided IL-2 for NK cell expansion in addition to low-dose exogenous IL-2. Apart from indirect inhibition of NK cells, Tregs can also directly suppress NK cells in a tumor microenvironment by secreting TGF-β, which is known to suppress NKG2D expression on NK cells and mitigate tumor cell lysis [44]. From these examples, it is clear that cross talk of NK cells with different components of the PBMCs plays a critical role in the regulation of NK cell proliferation and function.

In our study, NK cells infiltrated into small diffuse tumor nodules in the peritoneal cavity and were able to degranulate to induce cytotoxicity. This models residual tumor burden or diffuse persistent ovarian carcinoma where tumor sizes are small and perhaps not as immunosuppressive as compared to an advanced tumor. For the treatment of advanced tumors, NK cell therapy in combination with other treatment modalities may be required to achieve maximal clinical benefit. Various types of combination immunotherapies have been carried out in ovarian carcinoma patients, with promising results [45]. A new clinical trial is recruiting patients with recurrent ovarian and fallopian tube cancer along with other primary peritoneal carcinomas, for a phase1 study looking at the effect of *in vivo* suppression of T cells by using an indoleamine 2,3-dioxygenase (IDO) inhibitor, along with IP delivery of haploidentical NK cells (Clinicaltrials.gov identifier: NCT02118285). IDO is prevalent in ~56% of ovarian carcinomas and is associated with an immunosuppressive tumor microenvironment [46]. Also, in the ID8-VEGF ovarian carcinoma mouse model, decreased proliferation and the inability of the tumor infiltrating lymphocytes to produce cytokines was attributed to the expression of CTLA-4 and PD-1 makers [47]. Therefore, a combination of NK cell therapy with other strategies such as checkpoint inhibition may result in better clinical responses. For HLA expressing tumors that are resistant to NK cell lysis, combining NK cell therapy with an anti-KIR receptor antibody may be useful [48]. Because of the heterogeneous nature of tumors and a myriad of escape mechanisms used by tumors to evade the immune system, combining different approaches that inhibit mechanisms for immune escape in eclectic combinations is a rational approach.

NK cell function is regulated by a balance between various combinations of activating and inhibitory signals that allow the diversity of NK cell repertoire in an individual, but the link between NK cell diversity and NK cell function is only starting to be understood. In an analysis of peripheral blood NK cells, a study showed that there are 6-30,000 phenotypic variants of NK cells in an individual [49]. While inhibitory receptor expression is largely genetically governed, activation receptors are influenced by environmental factors in an individual [49]. The whole PBMC approach in our study rather than using enriched, *in vitro* IL-2 pre-activated NK cells, likely resulted in the conservation of the diversity of the activated NK cell repertoire due intercellular stimulation of NK cells with other components of the healthy donor PBMC population and tumor. This may have contributed to effectiveness of tumor induced NK cell expansion *in vivo* from low starting percentages (8-11%) to ~20% NK cells in the total lymphocyte population, and stimulation of anti-tumor efficacy. In adoptive transfer of isolated, pre-activated NK cells, the lack of intercellular stimulation from other lymphocyte types and potential skewing of NK cell population during *in vitro* pre-activation may possibly account for the lack of NK cell expansion and maximizing anti-tumor efficacy in ovarian carcinoma patients. For an effective anti-tumor response, all components are likely to have a role, but the absence of NK cells has shown to impair tumor rejection [50].

Results from these studies suggest that the cross talk of NK cells with other cell types in the PBMC population may be responsible for NK cell proliferation, infiltration into small and diffuse tumors and anti-tumor cytotoxicity. This may be an important consideration for utilizing NK cell-based immunotherapy as an adjuvant after surgical tumor resection and frontline chemotherapy, especially in the case of tumors where the elimination of residual tumor could be very significant to prevent relapse, such as in ovarian cancer. Therefore, this study provides a proof-of-principle for the therapeutic potential of NK cells in the anti-tumor response and highlights the importance of further investigating cell-to-cell interactions within PBMC population, based on which, different combination approaches could be designed to significantly attenuate tumor evasion.

## MATERIALS AND METHODS

### Animals

Animal care and use was at the Association for Assessment and Accreditation of Laboratory Animal Care International (AAALAC)-accredited University of Central Florida (UCF) vivarium. Protocols were approved by the UCF Institutional Animal Care and Use Committee (IACUC) and were compliant with NIH guidelines. NOD.Cg-*Prkdc^scid^ Il2rγ^tm1Wjl^*/SzJ (NSG) mice were obtained through an institutional material transfer agreement with Jackson Laboratory, Bar Harbor, Maine, USA.

### Human ovarian tumor cells and engraftment in mice

Human ovarian tumor cell line SKOV-3/GFP-Luc was obtained from Cell BioLabs, Inc. (San Diego, CA, USA). The cell line was passaged in an NSG mouse once, then sorted for GFP expressing tumor cells using a cell sorter (FACSAria, BD Biosciences, Franklin Lakes, NJ, USA). NSG mice were IP injected with 1×10^6^ SKOV-3-GFP/Luc cells and tumors seeded for 7-10 days. Tumor growth was monitored by *in vivo* bioluminescent imaging.

### *In vivo* imaging

SKOV-3/GFP-Luc tumor formation and growth was monitored using the Xenogen *In vivo* Imaging System (IVIS, Caliper Life Sciences, Hopkinton, MA, USA). Luciferin (Gold Biotechnology, Inc., St. Louis, MO, USA,) was IP injected at a dose of 0.15mg/g body weight fifteen minutes prior to imaging. Maximum/minimum of the luminescence signal intensity was adjusted to be the same throughout the experiment (Living Image software, Perkin Elmer, Waltham, MA, USA).

### Human PBMCs and *in vivo* treatments

Human PBMCs were obtained from healthy human donor source leukocytes (OneBlood, Orlando, FL, USA) by standard Ficoll gradient. Isolated PBMCs were tested for viability using Annexin V staining and NK cell percentages by flow cytometry using a BD Accuri C6, BD Biosciences, San Jose, CA, USA) following staining by CD45, CD3 and CD56 antibodies. The initial NK cell percentages for PBMCs used in the experiments were 8-11% of total CD45^+^ cells. After tumor engraftment (7-10 days after inoculation), 1 × 10^6^ viable PBMCs and 1,000 U human IL-2 (Preprotech, Rocky Hill, NJ, USA) were IP injected. 1,000 U IL-2 injections were given to mice weekly on Monday, Wednesday and Friday throughout the experiments. The ‘untreated’ mice in the experiments comprised of groups that were IP injected with 1) IL-2, 2) PBS or 3) were not treated with anything. All these mice behaved exactly the same with respect to the progression of the disease and their survival. Therefore, for the ease of representation, they are represented as one group and called as ‘untreated’ (with respect to PBMCs).

### Flow cytometry

Fifteen minutes after subcutaneous saline injections to hydrate the animals, <100 μL of peripheral mouse blood was collected by submandibular bleeds once a week. 50 μL of blood was treated with red blood cell lysis buffer and stained with fluorophore conjugated antibodies against hCD45 (eBiosciences, San Diego, CA, USA), hCD56 (Miltenyi Biotec, Bergisch Gladbach, Germany) and hCD3 (Beckman Coulter, Brea, CA, USA). Peritoneal washes obtained by flushing ~2mL PBS in the peritoneal cavity were also processed similarly (i.e., 50 μL of sample analyzed). Analysis of the data was done using the software Flowlogic (Inivai Technologies, Victoria, Australia). All NK cells (CD56^+^, CD3^−^) were first gated on human CD45^+^ cells to find percent NKs, and based on the total number of events shown on the plots and the total volume of blood analyzed, the “cells/mL” numbers were obtained (NK cells/mL = number of NK events/volume of blood analyzed).

### Histology

Tumors were fixed in 10% neutral-buffered formalin (Surgipath, Leica, Buffalo Grove, IL). Tissue was processed, embedded in paraffin blocks and 5 μm sections were stained. Immunohistochemistry (IHC) was done using Leica's BondMax automated immunostainer. Antibodies included NKG2D (GeneTex, Inc., Irvine, CA, USA). Granzyme B (Spring Bioscience, Pleasanton, CA, USA), Cleaved Caspase 3 (Cell Signaling Technology, Inc., Danvers, MA, USA), CD3 (Abcam Cambridge, MA, USA). The images shown in Figure 4 are from sequential sections from the blocks and represent the same peripheral region of the tumor, three dimensionally.

### Granzyme assay

From the tumors of mice treated for 14 days with PBMCs, cells were perfused using 18 gauge needles. They were washed and incubated with SKOV-3/GFP-Luc cells (50,000/well) on a 96-well plate for 1 hour. Cells were stained with anti-CD45, -CD3 and -CD56 antibodies in a granzyme substrate (PanToxiLux kit; Oncolmmunin, Gaithersburg, MD, USA). After 30 min incubation with antibodies, samples were analyzed using flow cytometry (CantoII, BD Biosciences) and Flowlogic software. To analyze the granzyme substrate signal (Fluorescein isothiocyanate, FITC) in the background fluorescence from the endogenous green fluorescent protein (GFP) of SKOV-3/GFP-Luc cells present in the NK cell (or T cell)-SKOV-3/GFP-Luc conjugates, a sample without granzyme substrate was used to define the fluorescent intensity for events correlating to cells that lack granzyme activity (Figure 4b).

## SUPPLEMENTARY FIGURES


