# Supplementary material for: Anti-ovarian tumor response of donor peripheral blood mononuclear cells is due to infiltrating cytotoxic NK cells
Source: Oncotarget. 2016 Jan 18;7(6):7318–28. doi: 10.18632/oncotarget.6939 (PMC4872788; doi:10.18632/oncotarget.6939)
Supplement: Supplementary file 1 [file oncotarget-07-7318-s001.pdf]

SUPPLEMENTARY FIGURES

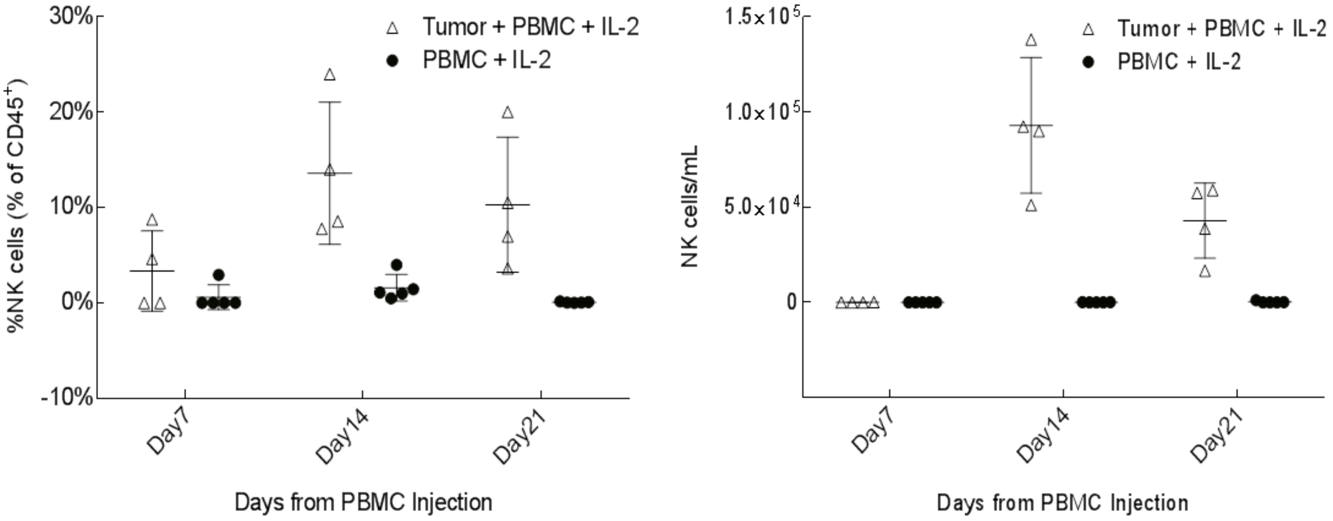

Supplementary Figure S1: Plots showing NK cell expansion and reduction expressed as a fraction (left) and as concentration (right), in peripheral blood. These were analyzed parallel to the data shown in Figure 3. p=0.07 (left); p=0.0001 (right) (two-way ANOVA).

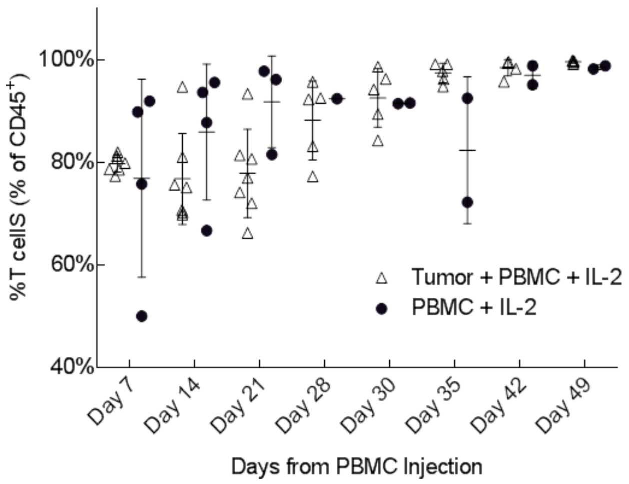

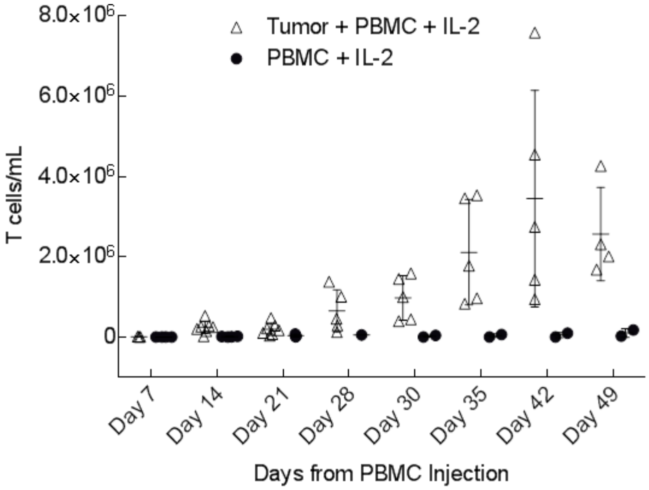

Supplementary Figure S2: Plots showing T cells in the peripheral blood over time in tumor bearing and non-tumor bearing mice treated with PBMC+IL-2, as a fraction (left) and as concentration (right). This data is from the survival experiment shown in Figure 1. p=0.01 (left); p=0.06 (right) (two-way ANOVA).

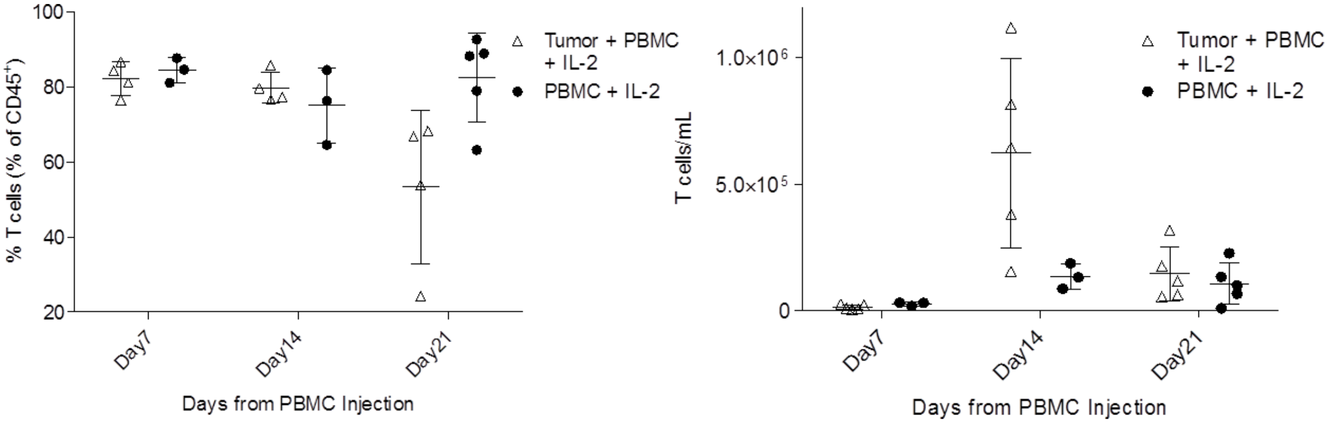

Supplementary Figure S3: Plots showing T cells in the peritoneal wash over time in tumor bearing and non-tumor bearing mice treated with PBMC+IL-2. This data is from the experiment shown in Figure 3. p=0.02 (left); p=0.01 (right) (two-way ANOVA).

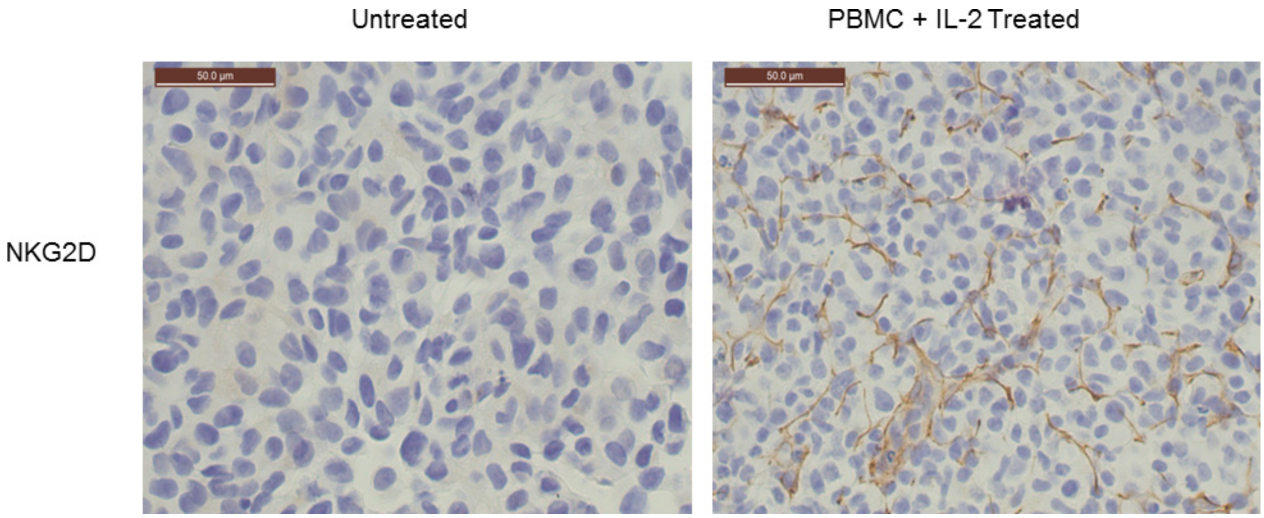

Supplementary Figure S4: IHC images showing NKG2D staining in ‘untreated’ and ‘PBMC +IL-2’ treated tumor tissue, indicating infiltration of NK cells into the tumors.

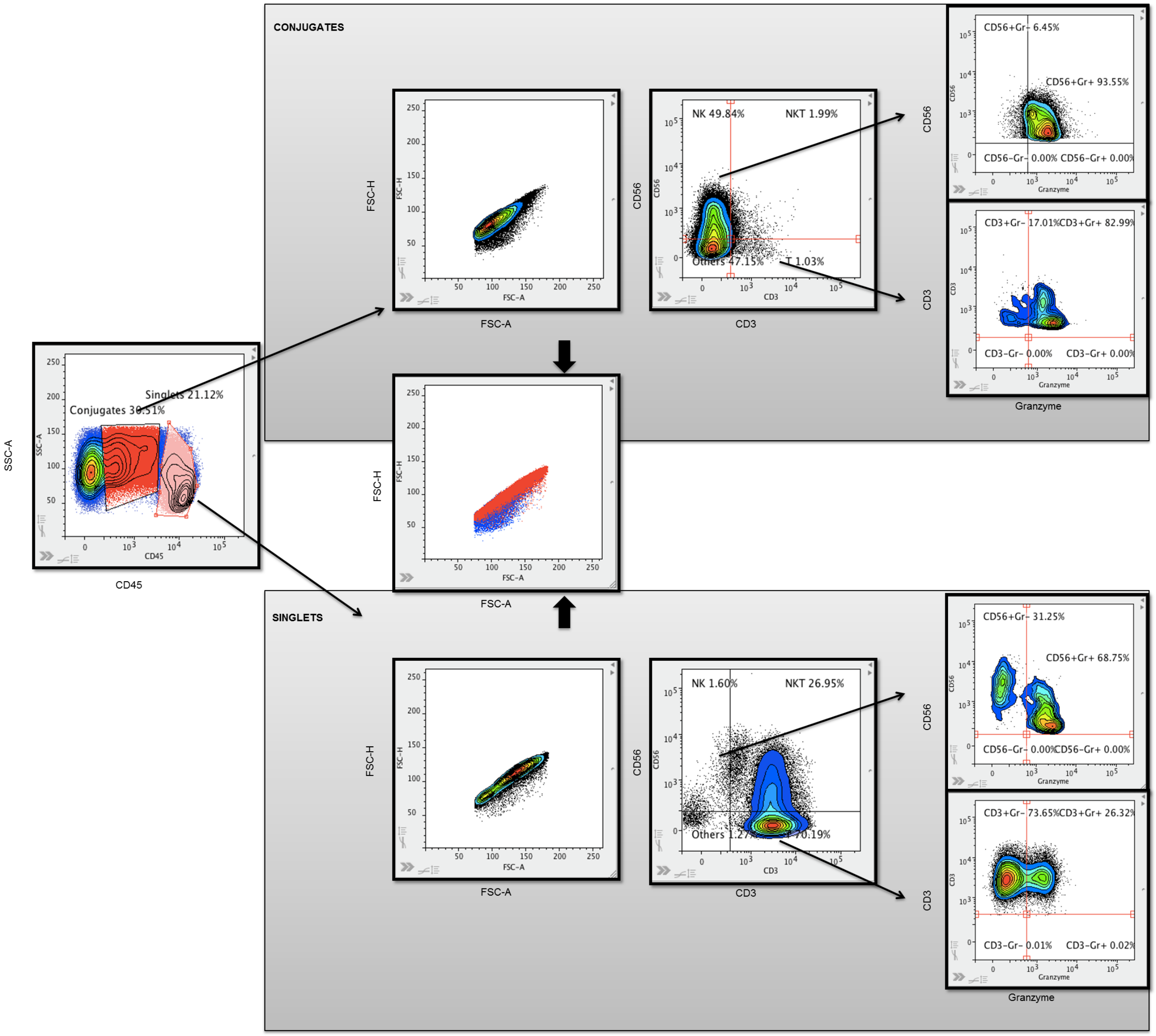

Supplementary Figure S5: Schematic of the gating strategy for the granzyme activity assay shown in Figure 5. First panel (left) shows the two distinct CD45^{+} populations, conjugates and singlets. In the second panel, FSC-H vs FSC-A plots for both populations are represented as overlays show conjugate formation. Third panel shows gating of the two populations for CD56 and CD3. The last panel shows granzyme activity positive events gated on the NK cell population**.**

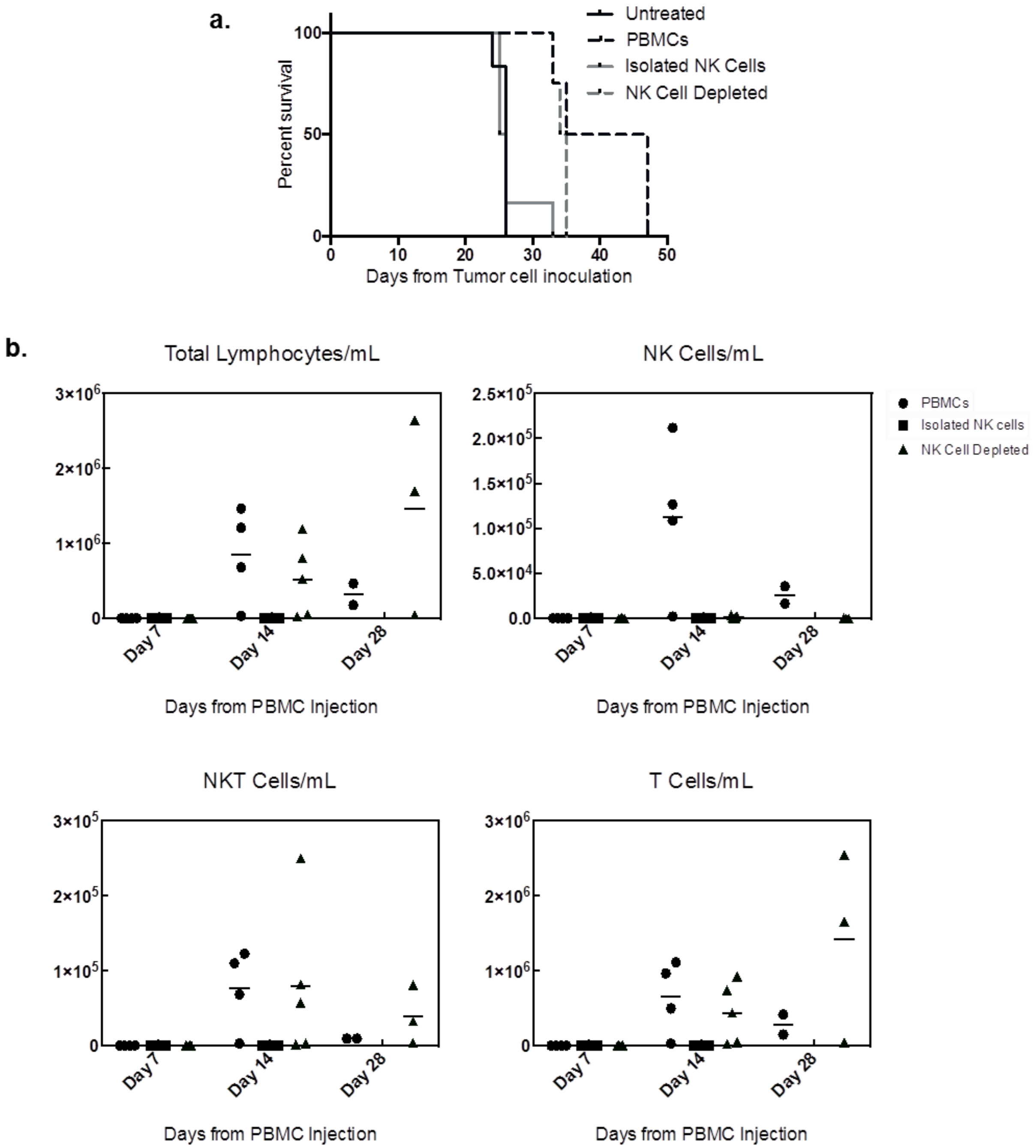

Supplementary Figure S6: a. Survival curve showing the differences in overall survival of the untreated, PBMC treated, isolated NK treated and NK depleted PBMC treated mice from the day of tumor cell inoculation. **b.** Plots showing the concentration of total lymphocytes ($CD45^+$), NK cells ($CD3^-$ $CD56^+$), NKT cells ($CD3^+$ $CD56^+$) and T cells ($CD3^+$ $CD56^-$) in the peripheral blood of the tumor bearing mice.
